# Transcriptome analysis and comparison reveal divergence between two invasive whitefly cryptic species

**DOI:** 10.1186/1471-2164-12-458

**Published:** 2011-09-22

**Authors:** Xiao-Wei Wang, Jun-Bo Luan, Jun-Min Li, Yun-Lin Su, Jun Xia, Shu-Sheng Liu

**Affiliations:** 1 Ministry of Agriculture Key Laboratory of Agricultural Entomology, Institute of Insect Sciences, Zhejiang University, Hangzhou 310029, China

## Abstract

**Background:**

Invasive species are valuable model systems for examining the evolutionary processes and molecular mechanisms associated with their specific characteristics by comparison with closely related species. Over the past 20 years, two species of the whitefly *Bemisia tabaci *species complex, Middle East-Asia Minor 1 (MEAM1) and Mediterranean (MED), have both spread from their origin Middle East/Mediterranean to many countries despite their apparent differences in many life history parameters. Previously, we have sequenced the transcriptome of MED. In this study, we sequenced the transcriptome of MEAM1 and took a comparative genomic approach to investigate the transcriptome evolution and the genetic factors underlying the differences between MEAM1 and MED.

**Results:**

Using Illumina sequencing technology, we generated 17 million sequencing reads for MEAM1. These reads were assembled into 57,741 unique sequences and 15,922 sequences were annotated with an E-value above 10^-5^. Compared with the MED transcriptome, we identified 3,585 pairs of high quality orthologous genes and inferred their sequence divergences. The average differences in coding, 5' untranslated and 3' untranslated region were 0.83%, 1.66% and 1.43%, respectively. The level of sequence divergence provides additional support to the proposition that MEAM1 and MED are two species. Based on the ratio of nonsynonymous and synonymous substitutions, we identified 24 sequences that have evolved in response to positive selection. Many of those genes are predicted to be involved in metabolism and insecticide resistance which might contribute to the divergence of the two whitefly species.

**Conclusions:**

Our data present a comprehensive sequence comparison between the two invasive whitefly species. This study will provide a road map for future investigations on the molecular mechanisms underlying their biological differences.

## Background

Genomic resources and information about invasive species are valuable for evolutionary studies to determine how and why phenotypes specific to non-indigenous species have been formed [1,2]. Moreover, they will aid ongoing efforts to understand and control the ecological, genetic and economic impacts of the invasive species. However, genomic or expressed sequence tag (EST) resources required to identify candidate genes or genomic changes associated with invasiveness are not yet developed for most invasive species [3]. In fact, only few invasive species have had significant genomic resources developed for this purpose [4]. Over the past several years, the next generation sequencing technology has significantly accelerated the speed of gene discovery and, is expected to boost genomics studies [5-7]. Because this technology eliminates the need for cloning ESTs, which introduces bias, and has greatly increased the quantity of data that can be generated in a short time at a reduced cost compared with traditional Sanger sequencing of cDNA libraries [8]. This technology has been proved to be a valuable addition to evolutionary and ecological research for non-model organisms [9]. However, so far, few studies have explored the potential of using next generation sequencing to investigate the source of genetic variation underlying the evolution of an invasive species [3,10].

The whitefly *Bemisia tabaci *(Gennadius) (Hemiptera: Aleyrodidae) has a global distribution with substantial genetic diversity and has been recorded over 600 species of plants [11-13]. Recent phylogenetic analysis combined with a determination of a consistent pattern of reproductive isolation among many genetic groups within *B. tabaci *indicates that the whitefly is a complex containing at least 28 cryptic species (herein species) [12,14-17]. Two species of the complex, Middle East-Asia Minor 1 (herein MEAM1) and Mediterranean (herein MED), as designated by Dinsdale *et al*. [15] and commonly referred to as the B and Q 'biotype' respectively in the past 20 years, have risen to international prominence since the 1980s due to their global invasion [18-22]. The invasive ability and damage potential of MEAM1 has earned it a place as one of the world's top 100 invasive species http://www.issg.org[23]. Some effort has been made to understand the multiple factors that contribute to the incursion of the two species into new regions and habitats. For example, asymmetric mating interactions between MEAM1 and its indigenous competitors have been shown to play a major role in the invasion of MEAM1 into China and Australia [19]. While both MEAM1 and MED are known for their invasiveness, their biological characteristics are rather different. For example, the invasion of MED seems more closely related to its strong resistance to major classes of insecticides [23-28]. Several studies have revealed that the greater abundance of MED relative to MEAM1 in Israel and southern Spain were associated with the higher levels of resistance to pyriproxyfen and neonicotinoids in MED [25,26]. Both species have been speculated to have a wide range of host plants, although up to date the knowledge of their actual host range is very limited partly due to the confusion of their species status in the past [12,29,30]. The experimental evidence available also shows clearly that the two species differ substantially in host range [31,32], interactions with begomoviruses [33,34] and mating behavior [35,36]. Because of those differences between MEAM1 and MED, competitive interactions between them where they co-occur are common and have significant impacts on their distribution. In Zhejiang Province of China, MEAM1 probably arrived in the late 1990s and has been rapidly displacing the indigenous species of the *B. tabaci *complex [19,37]. In 2005, MED appeared and gradually replaced MEAM1 and has become the only or predominant *B. tabaci *in some locations [37].

Natural selection under different ecological and agricultural environments is likely to have driven the evolution and divergence between MEAM1 and MED whiteflies and resulted in their reproductive isolation and biological differences. However, the molecular factors responsible for the differences between MEAM1 and MED species are almost unknown. Furthermore, we have no information about how natural selection may have affected the transcriptomes of these invasive whiteflies since their divergence from a common ancestor. So far, studies about sequences divergence in this whitefly species complex only focused on a few genes, such as cytochrome oxidase 1, nuclear ribosomal intergenic transcribed spacer 1, and 16S ribosomal DNA, which are important molecules to differentiate genetic groups of *B. tabaci *[12,15]. The sequence divergence of acetylcholinesterase among different whitefly populations of MEAM1 has also been documented because of its role in insecticide resistance [38]. However, the investigations of individual genes can not provide an accurate description of genome wide DNA sequence divergence. A more robust picture of genomics divergence between MEAM1 and MED may be attained by examining large numbers of genes that have been selected without prior interest in their biological functions or evolutionary histories [39]. The transcriptome represents a sample of the spatiotemporally expressed genome and can be used as an entry into the genome divergence study [40].

Previously, we have sequenced the transcriptome of MED using Illumina sequencing technology and demonstrated that this technology allows *de novo *transcriptome assembly in a species lacking genome information [41]. In this study, we generated over one billion bases of high-quality DNA sequence for MEAM1 with Illumina technology. Based on these DNA sequences, we identified 57,741 distinct sequences including hundreds of insecticide target and metabolism genes. To reveal the genetic differences between MEAM1 and MED, we compared the sequence variations between them and identified a number of orthologous genes that show signs of diversifying natural selection. The assembled, annotated transcriptome sequences of MEAM1 provide an invaluable resource for the identification of whitefly genes involved in biological invasion, insecticide resistance and host adaptation. The identification of divergent sequences between MED and MEAM1 opens the door for future investigation of the molecular mechanisms underlying the biological variations between them.

## Results

### Illumina sequencing and reads assembly of MEAM1 transcriptome

**Figure 1:**
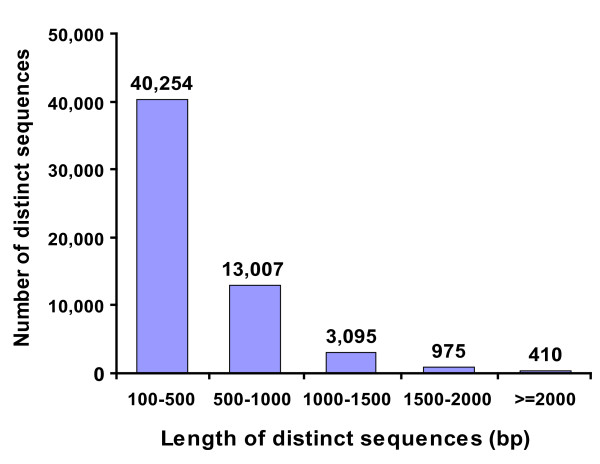
**Length distribution of distinct sequences**. The numbers of distinct sequences are shown on the top of each bar.

To obtain an overview of the MEAM1 whitefly transcriptome, a cDNA sample was prepared from a mixture of RNA from egg & nymph, pupa, female adult and male adult at equal ratio, and sequenced using the Illumina sequencing platform. We obtained a total of 17 million of 75 bp reads, which have been deposited in the NCBI Short Read Archive (SRA) under the accession number: SRX022878. These raw reads were assembled using SOAPdenovo software and resulted in 123,055 contigs (Table 1) [42]. The contigs were assembled into 104,722 scaffolds using paired-end joining and gap-filling (mean size: 326 bp) (Table 1). The 104,722 scaffolds were further clustered into 57,741 distinct sequences including 135 clusters and 57,606 singletons (Table 1). In this article, a cluster means a sequence composed of several scaffolds and the singleton means a scaffold that matches no other scaffolds. Next, we analyzed the length distribution of the 57,741 distinct sequences. As shown in Figure 1, although nearly 70% of the sequences (40,254) are between 100 to 500 bp, we identified 4,480 sequences longer than 1,000 bp.

**Table 1:** Summary for the MEAM1 whitefly transcriptome

Total number of reads	17,049,500
Total base pairs (bp)	1,278,712,500
Average read length (bp)	75
Total number of contigs	123,055
Mean length of contigs (bp)	269
Total number of scaffolds	104,722
Mean length of scaffolds (bp)	326
Clusters	135
Singletons	57,606
Total unique sequences	57,741
Sequences with E-value < 10^-5^	15,922

### Annotation of predicted proteins and Gene Ontology classification

**Figure 2:**
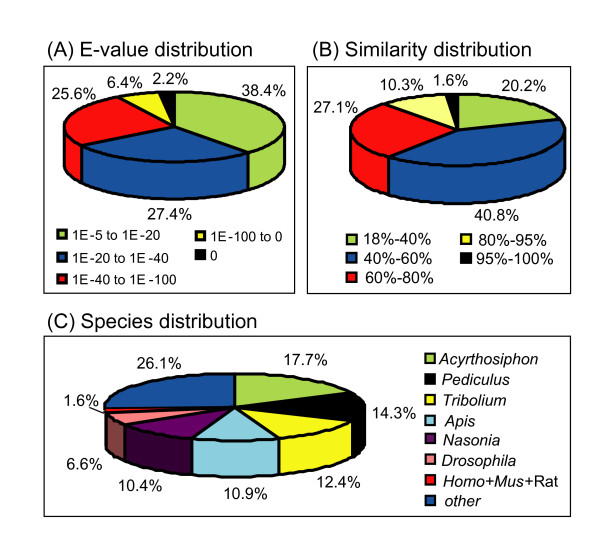
**Characteristics of homology search of assembled sequences against the nr database**. **A**. E-value distribution of best BLASTx hits for each distinct sequence with a cut-off E-value of 1.0E-5. **B**. Similarity distribution of the best BLAST hit for each sequence. **C**. Species distribution is shown as a percentage of the total homologous sequences with an E-value of at least 1.0E-5. We used the first hit of each sequence for analysis. Homo: *Homo sapiens*; Mus: *Mus musculus*; Rat: *Rattus norvegicus*.

For functional annotation, distinct gene sequences were searched using BLASTx against the non-redundant (nr) NCBI nucleotide database using a cut-off E-value of 1 × 10^-5^. A total of 15,922 genes returned an above cut-off BLAST result representing about 27.6% of all distinct sequences (See additional file 1). The E-value distribution of the top hits in the nr database showed that 34.2% of the mapped sequences have strong homology (smaller than 1.0E^-40^), whereas 65.8% of the homolog sequences ranged between 1.0E^-5 ^to 1.0E^-40 ^(Figure 2A). Likewise, the similarity distribution showed that 39% of the sequences have a similarity higher than 60%, while 61% of the hits have a similarity ranging from 18% to 60% (Figure 2B). Similar to the results of MED transcriptome [41], the highest percentage of MEAM1 sequences were matched to the pea aphid (*Acyrthosiphon pisum*) (17.7%), followed by the body louse (*Pediculus humanus corporis*) (14.3%), red flour beetle (*Tribolium castaneum*) (12.4%) and honey bee (*Apis mellifera*) (10.9%) (Figure 2C). Gene Ontology (GO) assignments were used to classify the functions of the predicted MEAM1 whitefly genes. Based on sequence homology, 4,711 sequences can be categorized into 52 functional groups under three main divisions (See additional file 2, red bars). Next, we compared the GO of MEAM1 and MED whitefly transcriptomes [41] and found that the distributions of gene functions from these two species are extremely similar (See additional file 2). This expected result indicates that there is no bias in the construction of the libraries from the MEAM1 and MED whiteflies. Compared to the MED transcriptome which has 7,330 sequences with GO annotation [41], the number of sequences with GO annotation in MEAM1 (4,771) is lower. This is probably due to the differences in the amount of sequencing data generated from the two samples (MEAM1: 1G; MED: 3G). For both species, in the three main divisions (cellular component, molecular function and biological process) of the GO classification, 'Cell part', 'Binding' and 'Cellular process', terms are dominant respectively. For both species, we also noticed a high-percentage of genes from categories of 'Cell', 'Catalytic' and 'Metabolic process' (See additional file 2).

### Identification and analysis of the orthologous genes between MED and MEAM1

**Figure 3:**
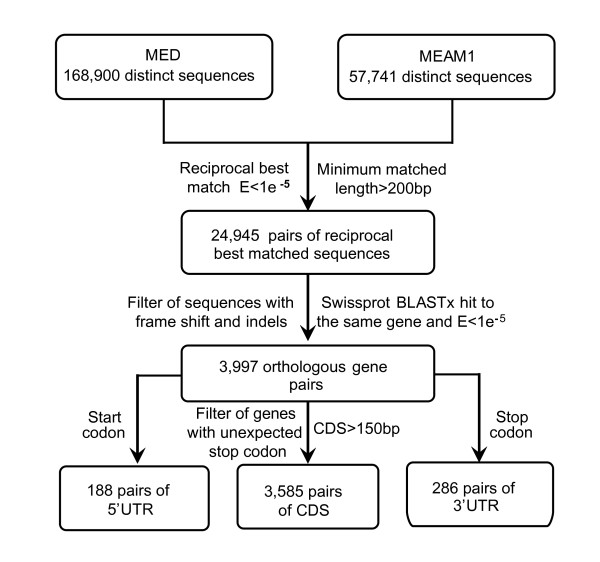
**Identification of the orthologous gene pairs between MEAM1 and MED**. The bidirectional best hit method was used to identify genes that are putatively orthologs. Coding sequences (CDS) of the orthologous genes were determined by BLASTx against all known proteins in Swissprot database using a threshold of 1.0E-5. After removing the UTR regions, sequences shorter than 150 bp and with unexpected codons in the CDS region were further filtered.

To compare the sequence divergence of the two species, we analyzed the possible orthologous genes between their transcriptomes using bidirectional best hit which has been widely used to identify orthologous genes [9,40,43,44]. By this way, we identified 24,945 pairs of putative orthologs with an average length of 397 bp and 98.22% identity (range from 80.2% to 100%). To remove potential paralogs, these putative orthologous genes were further screened against the Swissprot database. Only pairs of sequences that mapped unambiguously to the same protein in Swissprot database with an E-value < 1 × 10^-5 ^were selected as orthologous genes. Among these sequence pairs, 3,997 pairs of sequences could be mapped unambiguously to the same protein, suggesting strongly that they are orthologous genes (Figure 3). The untranslated region (UTR) of each sequence pair was identified based on the predicted coding region. Among the 3,997 pairs of orthologs, 188 pairs contain 5'UTR and 286 pairs contain 3'UTR. After removing the UTRs, we obtained the coding sequences (CDS) of all the orthologs. The CDS sequences containing unexpected stop codon and shorter than 150 bp were further filtered, resulting in 3,585 pairs of orthologous CDS sequences (Figure 3 and additional file 3). The average length of the 3,585 orthologous genes is 482.7 bp with an average similarity of 99.17%. The average GC content of orthologous CDS is 42.84% (Table 2), a value slightly higher than that of pea aphid genome (mean = 38.8) and *Apis mellifera *genome (mean = 38.6) [45,46]. However, the GC contents in the UTR regions of MEAM1 are 37.12% (5'UTR) and 35.19% (3'UTR) which is slightly lower than that of the pea aphid and honeybee genomes. Considering the large percentage of noncoding sequences in the whitefly genome [47], the overall GC content of the whitefly should be comparable to that of the pea aphid and honeybee. The 3,585 translated genes were annotated and classified using Kyoto Encyclopedia of Genes and Genomes (KEGG) database (See additional file 3). Given the differences between MEAM1 and MED, we propose that these pathways represent a transcriptome involved in core cellular and physiological functions common to the two whitefly species.

**Table 2:** Sequence divergence between the MED and MEAM1 cryptic species

				% Differences			
						
	% CpG	% GC	Loci	Mean	SE	Compared kb	ts/tv^e^
5' UTRs^a^	6.3	37.12	188				
All				1.66	0.153	21.11	1.41
No CpG				1.46	0.141	19.78	1.28
CpG				5.29	0.775	1.33	2.32
CDS^b^	6.57	42.84	3585				
All				0.83	0.014	1730.64	3.07
No CpG				0.61	0.011	1616.93	2.6
CpG				3.99	0.818	113.71	4.59
nd sites^c^	5.72	43.62	3585				
All				0.2	0.007	1024.65	1.34
No CpG				0.18	0.007	966.05	1.2
CpG				0.58	0.051	58.6	2.63
4d sites^d^	11.03	37.2	3585				
All				2.68	0.051	238.02	2.38
No CpG				1.73	0.041	211.77	1.69
CpG				12.16	0.332	26.25	3.94
3' UTRs	3.74	35.19	286				
All				1.43	0.114	43.1	1.56
No CpG				1.1	0.102	41.48	1.36
CpG				7.32	0.848	1.61	3.18

^a^UTRs: untranslated regions.

^b^CDS: coding sequence.

^c^nd sites: non-degenerative sites.

^d^4d sites: fourfold-degenerate sites where no changes cause any amino acid replacement.

^e^ts/tv: ration of transitions (ts) over transversions (tv).

### The sequence divergence between MEAM1 and MED

For the 5'UTR, the GC content is 37.12%, and 6.3% of the compared nucleotides occur in CpG contexts (Table 2). Differences between 5'UTRs of MED and MEAM1 orthologous genes occur at 1.66% of the positions. Interestingly, CpG sites in the 5'UTR differ at 5.29% of positions, whereas non-CpG sites differ at 1.46%. Thus, within 5'UTRs, differences occur approximately 3.6 times more often at CpG sites than at non-CpG sites. For the 3'UTR, the GC content is 35.19% and 3.74% of the nucleotides are in a CpG context. The overall difference of 3'UTR between MED and MEAM1 is 1.43%. CpG and non-CpG sites differ at 7.32% and 1.1%, respectively. Hence, in the 3'UTR, CpG sites contain 6.65 times more differences than non-CpG sites. These results suggest that a substantial proportion of the DNA sequence divergence between the two species is caused by changes at CpG sites. To understand the mechanism of evolution, we compared the ratio of transition (ts) and transversion (tv) [48]. Overall, the transitional differences are 1.41 times more frequent than transversional differences in 5'UTRs (Table 2). Interestingly, the transition-transversion ratio is higher in the CpG positions (2.32) than the non-CpG positions (1.28). This is consistent with the suggestion that the predominant type of mutations in the CpG sites is cytosine deamination, which results in transitional differences [49]. As in 5'UTR, transitional substitutions at 3'UTR are more common than transversions (ts/tv = 1.56) and transitions are even more frequent than transversions at CpG sites (ts/tv = 3.18, Table 2) compared with non-CpG sites (1.36). When comparing divergences of 3'UTR and 5'UTR, the overall and non-CpG sites divergence of 3'UTR is slightly lower than that at 5'UTR. However, for the CpG sites, the divergence at 3'UTR is higher than that of 5'UTR (7.32% versus 5.29%). Therefore, the mutation rates are likely to differ for CpG sites in different regions of genes. It reflects differences in mutational pressures between these regions because of differential methylation rate, which is also consistent with the finding that the transition/transversion rate is higher in the 3'UTRs than in the 5'UTRs (Table 2).

Among the 3,585 orthologous gene pairs, the overall divergence in coding regions is 0.83%. In non-CpG sites, the divergence is slightly lower (0.61%); whereas in the CpG sites, the divergence (3.99%) is 6.5 times as high as that of non-CpG sites. Apart from CpG context, the nucleotides in coding regions can further be classified as non-degenerative (nd) sites (any nucleotide substitutions produce amino acid change) and four fold degenerate (4d) sites (no changes cause amino acid replacement). From a total of 1,730 kb of coding region sequences, 1,025 kb are nd sites, whereas 238 kb are 4d sites (Table 2). At nd sites the overall divergence is 0.2%, whereas the overall divergence at 4d sites (2.68%) is 13.4 times that at the nd sites. The results indicate that the nd sites evolve under extensive functional constraints because any nucleotide substitutions at nd sites will produce amino acid changes. At nd sites, the GC content is 43.62% and the CpG content 5.72%. At nd sites, the non-CpG sites divergence between MED and MEAM1 is 0.18% and the divergence at CpG sites is 0.58% which is 3 times the non-CpG sites divergence. At the 4d sites, the GC content is 37.2%, whereas the CpG content is much higher (11.03%). At CpG sites, the divergence is 12.16% which is 7 times that at the non-CpG sites (1.73%). These results demonstrate that the higher percentage of divergence at the 4d sites is proportional to both the content of CpG sites and the rate of mutation.

### Synonymous and non synonymous sites

**Figure 4:**
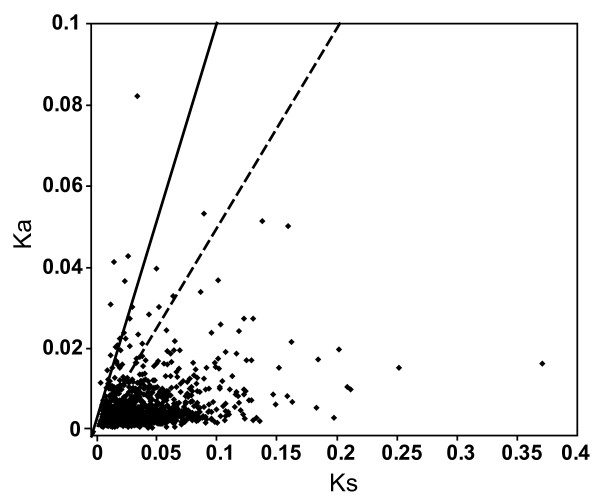
**Distribution of Ka and Ks**. Sequences with Ka/Ks > 1 fall above the solid line; while sequence with Ka/Ks between 0.5 -1 fall between the solid and dashed lines. Analysis was performed using the method of Yang & Nielsen (2000).

**Figure 5:**
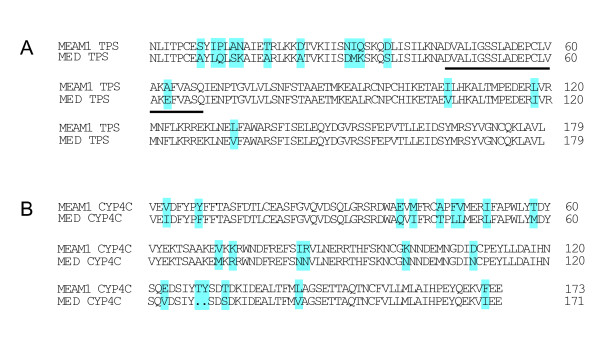
**Amino acid sequence alignments of alpha-trehalose-phosphate synthase and cytochrome P450**. **A**. Amino acid sequences alignment of the MEAM1 and MED alpha-trehalose-phosphate synthase (TPS). The putative catalytic domain is underlined. **B**. Amino acid sequences alignment of the MEAM1 and MED cytochrome P450 4C1 (CYP4C). The different amino acid residues are show in light blue.

To identify genes undergoing purifying and positive selections, we estimated rates of nonsynonymous (Ka) and synonymous (Ks) substitutions between MEAM1 and MED ortholog pairs. Among the 3,585 pairs of CDS, both a Ka and a Ks rate could be calculated for 1,161 orthologs (See additional file 4). For the rest of the gene pairs, we could calculate either only Ka or Ks. The 1,161 sequence pairs had mean values of Ka, Ks, and Ka/Ks of 0.0058, 0.0385, and 0.225. The Ka/Ks ratio has been widely used to measure the intensity and mode of selection under which a CDS is evolving. Ka/Ks > 1 is interpreted as a sign of positive selections and Ka/Ks < 1 is a signature of purifying selections [50-52]. Of the 1,161 sequence pairs with a Ka and a Ks rate, 24 orthologous gene pairs have a Ka/Ks value larger than 1 and 86 genes have a Ka/Ks value between 0.5 and 1 (Table 3 and Figure 4). Among the sequences with Ka/Ks values > 1, a couple of genes are involved in sugar and amino acids metabolic processes suggesting those processes are under strongly positive selection and are critical to the specialization of the whiteflies (Table 3). For example, the *alpha-trehalose-phosphate synthase *(TPS), which is a key enzyme for the synthesis of trehalose, has the highest Ka/Ks value (2.90). The amino acid sequences comparison of the TPS revealed that, in the MEMA1 species, a highly conserved glutamic acid residue in the catalytic domain is replaced by an alanine (Figure 5A) [53]. Except the sequences with high Ka/Ks, many sequences had Ka/Ks values equal to or only slightly greater than zero, suggesting that these genes have evolved under high selective constraint (See additional file 4) [39].

**Table 3:** List of genes with Ka/Ks larger than one

MEAM1 Gene IDs	S-Sub	N-Sub	Ka	Ks	Ka/Ks	Protein homolog
BT_B_ZJU_Singletons23470	16	2	0.0413	0.0142	2.9039	Alpha-trehalose-phosphate synthase
BT_B_ZJU_Singletons22749	5	1	0.0310	0.0117	2.6482	Hydroxyacylglutathione hydrolase
BT_B_ZJU_Singletons82918	16	3	0.0822	0.0342	2.4027	Oligopeptide transporter
BT_B_ZJU_Singletons9172	8	2	0.0429	0.0261	1.6434	RNA-directed DNA polymerase
BT_B_ZJU_Singletons22262	2	1	0.0148	0.0092	1.6172	Keratin 9
BT_B_ZJU_Singletons97129	5	1	0.0184	0.0115	1.5985	Diamine acetyltransferase
BT_B_ZJU_Singletons86701	8	2	0.0367	0.0238	1.5386	Putative ankyrin repeat protein
BT_B_ZJU_Singletons25715	4	1	0.0061	0.0045	1.3570	UPF0580 protein C15orf58 homolog
BT_B_ZJU_Singletons98955	4	1	0.0075	0.0057	1.3165	Coactivator of PPAR-gamma-like
BT_B_ZJU_Singletons14447	4	1	0.0063	0.0049	1.2796	Phosphatidylserine synthase 1
BT_B_ZJU_Singletons8887	4	1	0.0205	0.0162	1.2653	Cysteine proteinase
BT_B_ZJU_Singletons102961	4	1	0.0125	0.0100	1.2567	tRNA (cytosine-5-)-methyltransferase
BT_B_ZJU_Singletons103698	3	1	0.0104	0.0084	1.2325	tRNA (uracil-5-)-methyltransferase
BT_B_ZJU_Singletons103804	4	1	0.0116	0.0094	1.2288	Cyclin-K
BT_B_ZJU_Singletons69824	3	1	0.0210	0.0173	1.2133	Extracellular domains-containing protein
BT_B_ZJU_Singletons102115	16	4	0.0226	0.0192	1.1816	Retinol dehydrogenase
BT_B_ZJU_Singletons97093	3	1	0.0153	0.0130	1.1709	RNA-directed DNA polymerase
BT_B_ZJU_Singletons96386	3	1	0.0084	0.0076	1.1098	Mitogen-activated protein kinase organizer
BT_B_ZJU_Singletons102891	10	3	0.0120	0.0110	1.0931	Gamma-glutamyltranspeptidase 1
BT_B_ZJU_Singletons103844	3	1	0.0062	0.0058	1.0832	Serine/threonine-protein kinase PINK1
BT_B_ZJU_Singletons22561	3	1	0.0059	0.0055	1.0693	Uncharacterized protein
BT_B_ZJU_Singletons102559	7.74	2.26	0.0241	0.0226	1.0660	Cathepsin B-like cysteine proteinase 4
BT_B_ZJU_Singletons24476	3	1	0.0169	0.0166	1.0200	Krueppel homologous protein 1
BT_B_ZJU_Singletons15873	6	2	0.0304	0.0299	1.0162	Inactive purple acid phosphatase 29

The GeneIDs of the MED orthologs are lists in the Additional file 4.

S-Sub: Synonymous substitutions; N-Sub: Nonsynonymous substitutions; Ka: Nonsynonymous substitution rate; Ks: Synonymous substitution rate.

### Analysis of sequences with weak amino-acid similarity

To determine the transcriptome-wide levels of coding sequence divergence, we calculated the sequence homology of orthologous genes between MED and MEAM1. The 3,585 sequence pairs had a mean homology of 99.17% and ranged from 92% to 100% (See additional file 3). The large variation in sequence homology suggests that estimated values of species divergence based on few genes may produce misleading results. Among these sequence pairs, 604 show 100% homology, suggesting these genes are well conserved between MED and MEAM1 (See additional file 3). To reveal the proteins responsible for the differences between the two species, we analyzed the sequence pairs with weak amino-acid similarity. Interestingly, many of the divergent genes are related to sugar, protein and amino acid metabolism, such as *aminopeptidase *(92%), *oligopeptide transporter *(93.6%), *aspartate aminotransferase *(93.8%) and *galactose oxidase *(95.8%) (See additional file 3). The sequence divergences of these genes may cause functional differences in corresponding enzymes and result in the biological variations between MED and MEAM1. Furthermore, we noticed that a couple of proteins involved in insecticide resistance are also highly divergent, such as *cytochrome P450 4C1 *(93.4%) and *glutathione S-transferase *(95.3%). It was previously observed that the resistance to pesticides appeared to be enhanced by cytochrome P450 [54]. Therefore, we compared the protein sequence of the MED cytochrome P450 4C1 with that of MEAM1 (Figure 5B). Sequence alignment showed that the MED whitefly cytochrome P450 4C1 is clearly different with the MEAM1 homolog. The relevance of these mutations in pesticide resistance requires further investigation.

## Discussion

For the *B. tabaci *complex, 28 cryptic species have been delineated based on a number of phylogenetic analysis and reproductive isolation studies; however, the evolutionary origin and basal taxa have not been ascertained. Furthermore, the genetic factors associated with the evolution of invasive whitefly species are almost unknown. This is partially due to the fact that few molecules are available for inferring the evolutionary history and genetic divergence of *B. tabaci*. So far, only the *mitochondrial 16S DNA, cytochrome oxidase 1 *and the *nuclear ribosomal intergenic spacer *have been explored [12,15]. In this study, we have identified nearly 3,585 ortholog gene pairs and determined the average sequence divergence between MEAM1 and MED to be 0.83% for the CDS. This is much higher than the reported mean 0.45% divergence at the coding region between human and chimpanzee [55,56]. The gene divergence at the non-coding regions is even more obvious for 5'UTR (1.66%) and 3'UTR (1.43%) regions - nearly 1.5 times the divergence between the 5'UTR (1.12%) and 3'UTR (0.86%) regions of human and chimpanzee [56]. In addition, it is likely that our analyses might have underestimated the level of divergence between the two species. The orthologous genes were identified with very high stringency (reciprocal best match and the same unique Swissprot hit) which may have filtered out genes that have diversified in response to selection and no longer exhibit similarity significant enough to be identified with BLAST [39]. This strictness could have biased our data set toward more conserved sequences. Altogether, these results indicate that despite high-sequence identity in their CDS, the MEAM1 and MED species have diverged substantially between their transcriptomes. The level of sequence divergence provides additional support to the proposition that MEAM1 and MED are two species [15], in addition to the evidence of reproductive isolation [35,36].

Previous studies have shown that MEAM1 and MED differ in many life history parameters, such as mating behavior, insecticide resistance and host plant utilization [25,31,35,57]. Interestingly, we have identified a number of divergent sequences which might contribute to these biological differences between the two species. For example, MEAM1 and MED have different capacity to utilize various host plants and weeds [32,58]. In this study, we found that a number of genes involved in sugar and amino acid utilization are highly divergent, such as *alpha-trehalose-phosphate synthase, oligopeptide transporter, aminopeptidase *and *galactose oxidase*. Further functional characterization and comparison of these enzymes in MEAM1 and MED might reveal the molecular mechanisms underneath those differences. We also noticed that a couple of genes involved in insecticide resistance, such as *cytochrome P450 4C1 *and *glutathione S-transferase*, are clearly divergent between MEAM1 and MED. These differences might be responsible for the higher insecticide resistance of the MED species [25,54]. Nonetheless, the functions of those proteins in driving the evolution and divergence of the MEAM1 and MED species need to be further characterized.

The higher level of similarity observed in the CDS could be attributable to the presence of purifying natural selection on the CDS of the genes. To estimate the extent to which selection affects protein-coding DNA sequences, we calculated the number of nucleotide substitutions that change amino acids (Ka) and the number of substitutions that do not (Ks). Among the 3,585 pairs of transcript compared, the average Ka/Ks ratio is 0.225. Surprisingly, this ratio is remarkably similar to the average Ka/Ks ratio of rat and mouse coding region (0.19) and Ka/Ks ratio of human and chimpanzee (0.22) [56,59]. This is unexpected because the effective population size is much larger in whiteflies than in rodents and humans, which should have resulted in more effective selection against deleterious variants [60]. The ratio of Ka/Ks is a good indicator of selective pressure and has been used to identify protein-coding genes under positive and purifying selection [61]. Of the 1,161 orthologous pairs for which Ka and Ks could be calculated, 24 have Ka/Ks > 1, suggesting that those sequences might play important roles in the speciation and adaptive evolution of the whitefly. While the genes we have earmarked through this study are suitable subjects for future research, more rigorous statistical tests of positive selection, using multiple sequence samples, are required to confirm current results as well as to detect specific codons undergoing adaptive change.

Major advances in transcriptomics have become feasible in non-model organisms as a result of technology developments in next-generation sequencing. This study dramatically increased the number of genes from the MEAM1 whitefly (previously referred to as B biotype) [62]. Together with the MED whitefly transcriptome [41], we can provide an initial estimate of the number of transcribed genes in the whitefly. By reciprocal best match, we have identified 24,945 homologous sequences between MEAM1 and MED. Those 24,945 sequences were separately sequenced and assembled during the Illumina sequencing suggesting strongly that they are valid transcripts. As many distinct sequences represent nonoverlapping portions of the same transcript, this number is probably an overestimation of the actual genes and can be considered as an upper-bound of our library. For gene annotation, 12,931 sequences of the MEAM1 transcriptome have significant Swissprot hits with a minimum E-value of 1 × 10^-5 ^and 20,824 MED sequences have a significant hit [41]. As many of these Swissprot hits are likely to be duplicates (e.g., two sequences blasting to different parts of the same gene), we analyzed the unique gene names identified during these Blast searches. Among these Swissprot hits, 8,872 unique gene names were identified during the Blast search of the MEAM1 transcriptome and 11,219 unique gene names were identified for the MED transcriptome (data not shown). Considering both sets of results, the lower-bound of genes for the whitefly transcriptome should be more than 8,872. In fact, many assembled sequences lack matches to public database because they are either too short to permit appropriate alignments or represent highly divergent genes. For example, about 20% of the genes in the pea aphid (*Acyrthosiphon pisum*), the closest relative of *B. tabaci *with a sequenced genome, showed no homology to other metazoan genes [46]. Therefore, it is reasonable to postulate that the minimum number of genes in the whitefly will exceed 11,000. Although a precise estimation of transcriptome coverage is unachievable without the full genome information, our collection of unique transcripts represents a substantial percentage of the genes from the genome of *B. tabaci*.

## Conclusions

In this study, we have demonstrated that it is feasible to use Illumina sequencing to rapidly characterize multiple transcriptomes and compare their differences in an invasive non-model species. The level of sequence divergence is consistent with the previous proposition that MEAM1 and MED whiteflies are two species. Furthermore, we have identified hundreds of sequences showing high sequence divergence and found 24 genes under strong positive selection. The divergent sequences identified in this study will be an invaluable resource for studies of whitefly speciation, invasion, insecticide resistance and host plant utilization. To our knowledge, this is the first attempt using Illumina sequencing to study the transcriptome divergence of invasive species. We anticipate that this methodology holds great potential for the identification of genetic variation underlying the evolution of other invasive species.

## Methods

### Insect rearing and sample preparation

Cotton (*Gossypium hirsutum *cv. Zhe-Mian 1793) was cultivated to the 7-8 true-leaf stage for experiments. A pair of virgin adults of MEAM1 (mitochondrial cytochrome oxidase 1 gene GenBank accession no: GQ332577) were released onto cotton plant to oviposit and develop for five generations in a climate chamber at 27 ± 1°C, a photoperiod of 14 h light:10 h darkness and 70 ± 10% relative humidity [63]. The purity of the culture was monitored using the random amplified polymorphic DNA-polymerase chain reaction technique and the sequence of mitochondrial cytochrome oxidase 1 gene, which has been used widely to differentiate *B. tabaci *genetic groups [28]. Since the quantity of eggs was extremely low, a mixture of eggs and first to third instar nymphs were collected as one sample. The pupae were collected as another sample. For adults, individuals were collected from the culture using a glass tube (5 × 0.5 cm) and the sex was determined under a stereo microscope. Then the male and female adults were pooled separately into plastic tubes using an aspirator. Finally, these samples were frozen at -80°C until use.

### RNA isolation and library preparation for transcriptome analysis

Total RNA was isolated separately from the four samples (egg & nymph, pupa, female adult, and male adult) using SV total RNA isolation system (Promega) according to the manufacturer's protocol [64]. RNA integrity was confirmed using the 2100 Bioanalyzer (Agilent Technologies) with a minimum RNA integrated number value of 8. mRNA was purified from 8 μg of total RNA (a mixture of RNA from egg & nymph, pupa, female adult and male adult at equal ratio) using oligo (dT) magnetic beads. The cDNA library for transcriptome sequencing was prepared using Illumina's kit following manufacturer's recommendations as described before [41].

### Analysis of Illumina sequencing results

The cDNA library was sequenced at The Beijing Genome Institute (Shenzhen, China) on the Illumina sequencing platform (GAII). The size of the library is approximately 200 bp and both ends of the library are sequenced with a length of 75 bp. Image deconvolution and quality value calculations were performed using the Illumina GA pipeline 1.5. The raw reads were cleaned by removing adaptor sequences, empty reads and low quality sequences (reads with unknown sequences 'N'). The reads obtained were randomly clipped into 21 bp K-mers for assembly using de Bruijn graph and SOAPdenovo software [42]. After sequence assembly, the resultant contigs were joined into scaffolds using the read mate pairs. To obtain distinct gene sequences, the scaffolds were clustered using TGI Clustering tools with the default parameters [65]. Distinct sequences were used for Blastx search and annotation against the NCBI nr protein database using an E-value cut-off of 10^-5^. Functional annotation of GO terms http://www.geneontology.org was analyzed by Blast2go software. The data sets of Illumina sequencing are available at the NCBI Short Read Archive (SRA) with the accession number: SRX022878. The assembled sequences have been deposited in the NCBI's Transcriptome Shotgun Assembly (TSA) database under the accession number of HP643344 to HP701084 and can be searched using the GeneID listed in Additional file 1.

### Identification of orthologous genes and prediction of the coding and untranslated regions

We used the bidirectional best hit method in MegaBLAST to identify genes that are putatively orthologs between MEAM1 and MED. Pairs of sequences that were each other's best hit and longer than 200 bp were retained as putative orthologs. Bidirectional best hit has been widely used to identify the orthologous genes between closely related species [9,40,44] and this approach has been found to outperform more complex orthology identification algorithms [43]. As *de novo *transcriptome assemblies generally struggle to differentiate members of gene families, using bidirectional best hit to identify orthologs may not completely exclude the orthologous paralogs. Therefore, these putative orthologous genes were further screened against the Swissprot database to remove potential paralogs. If two sequences are orthologous paralogs, during the Blast search, they probably will hit to different genes in the Swissprot database. Only pairs of sequences that mapped unambiguously to the same protein in Swissprot database with an E-value < 1 × 10^-5 ^were selected as orthologous genes. Alignments containing any frameshifts and indels were filtered. CDS of the orthologous genes were determined by BLASTx against all known proteins in Swissprot database using a threshold of 1 × 10^-5^. CDS with unexpected stop codon in the Blast hit region and shorter than 150 bp were removed. Start codon positions were determined by examination of the in-frame ATG codon present 30 bp upstream or downstream of the beginning of the aligned reference protein. The 5'UTR of each pair of orthologs was identified based on the results of CDS and start codon prediction. The stop codon positions were determined by examination of in-frame TAA, TAG and TGA motifs present within 30 bp of the stop codon of the reference protein. Similarly, the 3'UTR of orthologs was defined based on the CDS and stop codon prediction. To prevent false positive results, only UTR pairs with an E-value < 1 × 10^-30 ^were selected for further analyses.

### Sequence divergence analyses and estimation of substitution rates

For each pair of orthologs, 5'UTR, coding and 3'UTR regions were extracted respectively. For the CDS region, pair-wise alignments were generated for all the orthologous gene pairs based on protein sequences and back-translated to DNA sequences for subsequent analysis. The CDS and UTR regions were aligned separately to each other with a MegaBlast algorithm and checked manually for errors. Alignments are available upon request. Because MEAM1 and MED are closely related, multiple substitutions at the same site are highly unlikely. Therefore, the sequence divergence was calculated by dividing the number of substitutions by the number of base pairs compared. The divergence was determined for the contexts of nondegenerate (nd), fourfold degenerate (4d), CpG and non-CpG [56]. The ratio of transitions over transversions (ts/tv) was determined for the 5'UTR, CDS and 3'UTR as well. Substitution rates were estimated separately for synonymous (Ks) and non-synonymous sites (Ka) using an approximate method implemented in the KaKs Calculator Version 1.2 [66]. Pair-wise approximate analyses were performed using the YN method [61]. Because the sequencing errors are distributed among synonymous and nonsynonymous sites at equal frequencies, they are not expected to influence the results of analyses [39].

## Authors' contributions

XWW, JBL and SSL conceived and designed the experimental plan. JBL, JML and YLS performed experiments. XWW, JML, YLS, JX and SSL analyzed and interpreted the sequence data. XWW, JBL, YLS and SSL drafted the manuscript. All authors read and approved the final manuscript.

## Supplementary Material

Additional file 1 **Top BLASTx hits from NCBI nr database**. BLASTx results against the NCBI nr database for all the distinct sequences with a cut-off E value above 1.0E-5 are shown. Click here for file

Additional file 2 **Histogram presentation of Gene Ontology (GO) classification of genes from the MEAM1 and MED whiteflies**. The results are summarized in three main categories: biological process, cellular component and molecular function. The right y-axis indicates the number of genes in a category. The left y-axis indicates the percentage of a specific category of genes in that main category. GO analysis showed that the distributions of gene functions for MEAM1 and MED whiteflies are similar. Click here for file

Additional file 3 **List of the orthologous gene pairs between MEAM1 and MED**. The length of orthologous region, sequence homology and Swissprot, nr, KEGG annotations were shown as well. Click here for file

Additional file 4 **Summary of Ka and Ks values of each orthologous gene pairs**. S-Substitutions: synonymous substitutions; N-Substitutions: nonsynonymous substitutions; Ka: nonsynonymous substitution rate; Ks: synonymous substitution rate. Click here for file
