# Supplementary figures and images for: Transcriptome analysis and comparison reveal divergence between two invasive whitefly cryptic species
Source: BMC Genomics. 2011 Sep 22;12:458. doi: 10.1186/1471-2164-12-458 (PMC3189941; doi:10.1186/1471-2164-12-458)

# Gene Ontology analysis

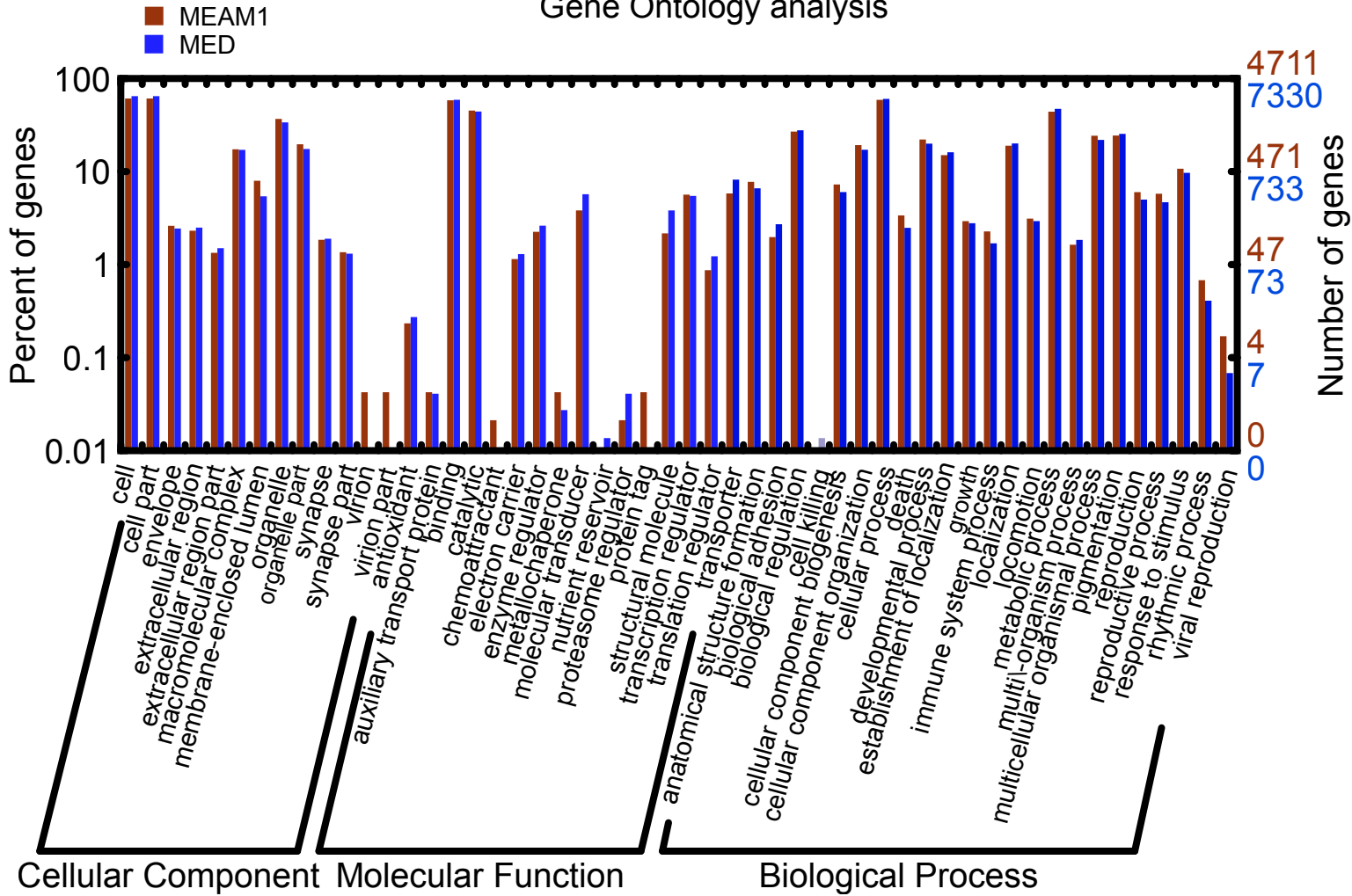

Supplement: Additional file 2 — Histogram presentation of Gene Ontology (GO) classification of genes from the MEAM1 and MED whiteflies. The results are summarized in three main categories: biological process, cellular component and molecular function. The right y-axis indicates the number of genes in a category. The left y-axis indicates the percentage of a specific category of genes in that main category. GO analysis showed that the distributions of gene functions for MEAM1 and MED whiteflies are similar. [file 1471-2164-12-458-S2.PDF]
